# Impact of two different periodized aerobic training on acute cerebrovascular response and cognitive performance in coronary heart disease patients

**DOI:** 10.14814/phy2.70211

**Published:** 2025-02-04

**Authors:** Béatrice Bérubé, Maxime Boidin, Mathieu Gayda, Thomas Vincent, Jonathan Tremblay, Martin Juneau, Anil Nigam, Antony D. Karelis, Louis Bherer

**Affiliations:** ^1^ Research Center and EPIC Center Montreal Heart Institute Montreal Québec Canada; ^2^ Research Center Institut Universitaire de Gériatrie de Montréal Montreal Québec Canada; ^3^ Department of Psychology Université du Québec à Montréal Montreal Québec Canada; ^4^ School of Kinesiology and Exercise Science, Faculty of Medicine Université of Montréal Montreal Québec Canada; ^5^ Department of Medicine, Faculty of Medicine Université of Montréal Montreal Québec Canada; ^6^ Liverpool Centre for Cardiovascular Science, Liverpool Heart and Chest Hospital University of Liverpool, and Research Institute for Sport and Exercise Sciences of Liverpool John Moores University Liverpool UK; ^7^ Department of Sport and Exercise Sciences Institute of Sport, Manchester Metropolitan University Manchester UK; ^8^ Department of Exercise Science Université du Québec à Montréal Montreal Québec Canada

**Keywords:** acute exercise, cerebral oxygenation, chronic exercise, cognitive functions, coronary heart disease

## Abstract

The aim of this study was to measure the effects of chronic and acute aerobic exercise at two different intensities on cognitive performance and cerebrovascular response in coronary heart disease (CHD) patients. Thirty‐five CHD patients completed two exercise bouts at 30% and 70% of their respective peak aerobic power on an ergocycle while performing cognitive tasks, which included nonexecutive and executive conditions before and after a 3‐month training intervention. Variations of oxy‐ deoxy‐ and total hemoglobin concentrations were measured on the left prefrontal cortex at both intensities using near‐infrared spectroscopy. Aerobic exercise training consisted of linear and nonlinear periodization protocols for three sessions of 30–50 min per week for 12 weeks. Error rate (*p* < 0.001) and reaction time (*p* < 0.001) improved after the training program for the executive condition of the cognitive task, regardless of intensity and training groups. Cerebral oxygenation remained similar pre and post intervention for all conditions and acute exercise intensity. Despite the absence of conjunction between cerebral oxygenation and cognition, results suggest that both exercise training programs could improve cognition in CHD patients during acute exercise.

## 1 INTRODUCTION

Acute aerobic exercise is often used as a stress test in clinical practice with cardiac patients to detect cardiovascular and cerebrovascular abnormalities (Alosco et al., 2013; Anazodo et al., 2015). In the context of cognition, the effects of an acute aerobic exercise could reflect critical situations of daily life such as housekeeping or walking (Ainsworth et al., 2000). Subsequently, studying cognitive function during a single bout of aerobic exercise could help detect potential dysfunctions that would be undetectable at rest, and identify possible physiological mechanisms involved in the heart–brain axis such as cardiorespiratory fitness.

### 1.1 Cognitive function at high intensity

At the brain level, a single bout of aerobic exercise leads to acute changes in cerebral blood flow (CBF) and cerebral perfusion, which could ultimately influence cerebral function (McMorris et al., 2016). High intensity acute aerobic exercise (≥80% of peak power output [PPO]) leads to a reduction in CBF, and impacts negatively executive performance and cerebral perfusion independently from age in healthy individuals compared to lower intensities (≤80% of PPO) (Ando, 2016; Labelle et al., 2013; Mekari et al., 2015). However, cognitive performance in lower fit healthy individuals was more affected by high intensity compared to higher fit counterparts (Labelle et al., 2013), supporting the notion that the effect of acute exercise on cognition may be more prominent in individuals with lower cardiorespiratory fitness such as at‐risk individuals. This difference between fit and unfit individuals might be explained by an unbalanced between the regulatory factors (such as blood pressure, cerebral metabolism, cardiac output, and the partial pressure of arterial carbon dioxide) (see review of (Smith & Ainslie, 2017)). In coronary heart disease (CHD) patients, there is evidence to suggest that the reduction in cerebral oxygenation and perfusion may be more pronounced at higher workloads (80% of maximal capacity and over) (Koike et al., 2004, 2008). Moreover, at 70% of PPO, CHD patients exhibited lower total cerebral perfusion in the left prefrontal cortex compared to aged‐match healthy controls, but executive performance (accuracy and reaction time) remained stable (Bérubé et al., 2021). This suggests that the intensity of exercise could affect cerebral oxygenation and perfusion differently, but not executive functions.

### 1.2 Training load variation and progression

In CHD patients, an aerobic exercise program should be progressive (i.e., linear periodization [LP]) and adapted to the patient‘s capacity and preference (Balady & Gardner, 2013). LP led to greater benefits in terms of oxygen uptake (V̇O_2_) improvement (de Macedo et al., 2018), and functional capacity (de Macedo et al., 2012) in CHD patients compared to no periodization. However, constantly increasing the training load is not realistic and could lead to fatigue and overreaching. Nonlinear periodization (NLP) is a novel way to incorporate variation in the training load to avoid a plateau and prevent overtraining and injuries (Blanchard & Glasgow, 2014). Greater benefits in cognitive performance have been observed after a NLP program compared to a LP in different clinical population such as overweight individuals (Ahmadizad et al., 2014), in patients with chronic pulmonary disease (Klijn et al., 2013), but similar benefits in patients with CHD (Boidin et al., 2020). However, improvement in cognitive performance seems to be dependent on the exercise parameters such as intensity and duration, rather than change in peak V̇O_2_. For example, healthy elderly individuals who trained at a greater physical activity intensity demonstrated greater cognitive performances (Brown et al., 2012). Thus, manipulating intensity and duration could be the target for improving cognitive performance rather than aiming an increase in peak V̇O_2_ in patients with CHD.

Thus, the aim of this study was to compare the effect of NLP to LP on acute exercise response on executive functions and cerebral oxygenation in CHD patients. We hypothesized that CHD individuals in the NLP group would show better cognitive performances and cerebral oxygenation in an acute exercise session compared to the LP group.

## 2 METHODS

### 2.1 Study design and participant's recruitment

The rationale and detailed study design of this study has been published previously (Boidin et al., 2020). Written informed consent was obtained from all participants prior to their enrollment in the study, and the study protocol was approved by the Research Ethics and New Technology Development Committee of the Montreal Heart Institute and registered on ClinicalTrials.gov (identifier number: NCT03443193). Briefly, 44 patients with stable CHD were enrolled at the Cardiovascular Prevention and Rehabilitation Center (EPIC) of the Montreal Heart Institute from December 2015 to September 2017, 40 patients with CHD completed the main randomized control trial study and 35 were included in this study. Five participants were excluded because of noisy near‐infrared spectroscopy (NIRS) signals. Exclusion criteria for CHD patients were the following: a recent acute coronary syndrome (<3 months); heart failure; left ejection fraction <40%; severe CHD non‐suitable for revascularization; scheduled coronary artery bypass surgery for severe coronary heart disease; chronic atrial fibrillation; malignant arrhythmias during exercising; restriction to cardiopulmonary exercise testing or severe intolerance to exercise.

### 2.2 Experimental design

At baseline and study‐end, all participants reported on three occasions to undergo testing, separated by at least 72 h, within a 14‐day period. During the first visit, participants underwent a medical evaluation including medical history, physical examination by a cardiologist, body composition, and anthropometric measures, and the Mini‐Mental State Evaluation (MMSE) and the Geriatric Depression Scale (GDS). Upon their second visit, all participants performed a maximal cardiopulmonary exercise testing (CPET) to measure peak V̇O_2_ and PPO on a cycle ergometer with gas exchange analysis (Boidin et al., 2020). During the third visit, participants completed the cognitive task session and cerebral hemodynamic response measurement using NIRS system during submaximal aerobic exercise (30% and 70% of the PPO in random order) (Bérubé et al., 2021). All participants were instructed to avoid eating 3 h prior to testing as well as to avoid alcohol and caffeine consumption 12 h prior to visits 2 and 3.

### 2.3 Measurements

#### 2.3.1 Maximal cardiopulmonary exercise testing

A CPET was performed on a cycle ergometer (Ergoline 800S, Bitz, Germany) using an individualized ramp protocol (3‐min warm up at 20 W, followed by an initial power of 20–25 W and increase in power output of 10–15 W.min^−1^ until exhaustion, while maintaining cadence >60 rotations per minute (rpm), followed by 2 min of active recovery at 20 W with a cadence at <60 rpm and 3 min of passive recovery) (Boidin et al., 2020; Gayda et al., 2017). The highest oxygen uptake value reached during a consecutive 30‐s period was considered as the peak V̇O_2_. The PPO was defined as the power output reached at the last completed stage. Continuous electrocardiogram (ECG) monitoring (Marquette, case 12, St. Louis, Missouri), rating of perceived exertion (RPE: Borg Scale, 6–20), and manual blood pressure using a sphygmomanometer (Welch Allyn Inc., Skaneateles Falls, USA) were monitored throughout the test every 2 min. Strong verbal encouragements were given throughout the test.

#### 2.3.2 Computerized modified Stroop task

The computerized modified‐Stroop task was based on previous work that used acute exercise and cognition including nonexecutive (naming) and executive (inhibition and switching) during experimental conditions (Bérubé et al., 2021; Labelle et al., 2013; Mekari et al., 2015). Both conditions included two nonexecutive and two executive block repetitions. Each sequence of the test session lasted 8 min and was composed of 1‐min blocks for each cognitive condition separated by a 1‐min rest block. Each block began with a fixation cross (XXXX) for 500 ms followed by the 15 visual stimulus trials which appeared on the screen for 2500 ms. Naming blocks consisted of 15 trials showing neutral‐colored (blue or green) visual stimuli (i.e., fixation cross, XXXX). Executive blocks also included 15 trials, which were divided in two types: inhibition and switch trials accounting for 75% and 25% of the total number of trials, respectively. Switch trials appeared randomly throughout the executive block. Error rate (percentage of errors from the total trial, %) and reaction time were collected for all trials.

#### 2.3.3 Submaximal acute aerobic exercise protocols

Participants completed a submaximal test on an ergocycle at constant power output of 30% and 70% of their respective PPO (Bérubé et al., 2021). Exercise intensity levels were equally counterbalanced across participants among both groups to avoid a systematic association (Winer, 1971). Participants started with the Stroop task practice at rest followed by a 2‐min rest state, then a 3‐min warm‐up at 20 watts followed by a 2‐min phase to reach the first power steady‐state (30% or 70% of PPO). An 8‐min constant bout of exercise while Stroop task was performed until the end this sequence was then completed. Participants rested for 4 min before starting the second block. A 2‐min active recovery at 20 watts and a 3‐min passive recovery followed the second bout of acute exercise. Partial pressure of end‐tidal carbon dioxide (P_ET_CO_2_) was taken from the CPET data by averaging all the closest data of 30% and 70% of PPO (Nassar & Schmidt, 2017).

#### 2.3.4 Cerebral hemodynamic response

Cerebral oxygenation and perfusion were indirectly measured by the changes in relative concentration (ΔμM) of oxyhemoglobin ([HbO]) and deoxyhemoglobin ([HbR]), and obtained using a noninvasive continuous‐wave NIRS system (Oxymon Mk III, Artinis Medical, Netherlands) (Bérubé et al., 2021). Optodes were placed at the level of the left prefrontal cortex in Fp1 and Fp3 (Davenport et al., 2012; Ekkekakis, 2009; Mehagnoul‐Schipper et al., 2002). NIRS data analysis was performed in Nirstorm, a plugin of Brainstorm (Tadel et al., 2011). To isolate the cognitive effect of acute exercise, baseline signals (exercise only) were subtracted from those recorded during the Stroop task performed while cycling. Movement artifacts were tagged for automatic correction (Scholkmann et al., 2010). Signals were then converted in optical density and a high pass‐band filter was applied to remove physiological artifacts. Signals were projected on the Colin27 template. The modified Beer–Lambert law allowed calculation for relative [HbO], [HbR] while total hemoglobin concentration ([HbT]) was obtained by the summation of [HbO] and [HbR], which was used as a measure of cerebral blood volume (CBV) (Ekkekakis, 2009). Extraction of within‐subject hemodynamic responses for each PPO level and task was obtained using a general linear model and normalized effects were calculated (Ye et al., 2009).

#### 2.3.5 Exercise training

All patients completed three‐weekly training sessions on cycle ergometer for a total of 36 sessions. Exercise training sessions consisted of a combination of short to medium stages of high‐intensity interval training (HIIT) and moderate intensity continuous training (MICT). In both protocols, the first 4 weeks included one HIIT and two MICT sessions, while two HIIT and one MICT session a week were performed from week 5 to 12 (Boidin et al., 2020; Ribeiro et al., 2017). Training load was constantly and progressively increased by 5% each week in the LP group. Although, training load increased by 8% each week for the first 3 weeks followed by a 5% decrease the fourth for the NLP group. This training cycle was repeated until the end of the training program. Following each aerobic training session, patients in both groups performed a similar non‐periodized resistance training program including six different exercises using elastic bands for every muscle groups.

### 2.4 Statistical analysis

Dependent variables of interest were error rate, reaction time, [HbO], [HbR], and [HbT]. Analyses were performed with SPSS 26 (IBM, United States). After ensuring a normal distribution with the skewness and kurtosis indexes (Curran et al., 1996), a two‐way mixed analyses of variance (ANOVAs) were performed with time (pre and post intervention) and groups (LP and NLP) for all dependant variables. Intensity order of exercise bouts did not interact significantly (all, *p* > 0.05) with our analyses, and there were no group differences on dependent variable of interests. Thus, intensity order was removed and not used as a covariate and both intensity order groups were combined. Greenhouse–Geisser correction was applied if violation of sphericity occurred. Post hoc analyses were conducted with pairwise comparisons using a Bonferroni correction (Ye et al., 2009). Adjusted *p*‐values and effect sizes (*η* ^2^) are reported. The magnitude of the effect sizes were interpreted as follow: small (*η* ^2^ = 0.01 to 0.08) medium (*η* ^2^ = 0.09 to 0.24) and large (*η* ^2^ = 0.25 and over) effects (Tabachnick & Fidell, 2007).

## 3 RESULTS

### 3.1 Demographics, global cognitive functioning, and physiological measurements

Table 1 shows demographics, neuropsychological, and psychological data for both groups. Five patients were excluded for the final analysis because of bad NIRS signals. Subsequently, a total of 35 participants were included in the final analysis. Both groups had high adherence rate for training participation (98%). There were no significant differences for the years of education, age, sex proportion, MMSE, and GDS scores between groups. There was no significant interaction between time and groups for estimated P_ET_CO_2_ at 30% and 70% of PPO.

**TABLE 1:** Demographic, neuropsychological, psychological, and physiological characteristics before and after the linear and nonlinear training interventions.

	Linear (*n* = 18)		Nonlinear (*n* = 17)		Interaction intervention*time	Main effect
	M (SD)		M (SD)			
	Pre	Post	Pre	Post	*p* Values	*p* Values
Demographics						
Age (years)	64 (10)	–	67 (6)	–	–	0.45
Sex (male/female)	14/4	–	14/3	–	–	>0.99
Education (years)	14.9 (3.8)	–	14.9 (3.6)	–	–	>0.99
Depression symptomatology						
GDS	6.5 (5.6)	6.5 (5.4)	5.5 (5.4)	5.4 (5.5)	0.89	0.89
Cognitive functioning screening						
MMSE	28.3 (1.3)	28.7 (1.4)	28.2 (1.3)	28.5 (1.6)	0.96	0.11
Physiological measurements						
Peak V̇O_2_ (ml.kg^−1^.min^−1^)	22.0 (5.5)	23.7 (5.9)	22.5 (5.2)	23.7 (5.7)	0.65	<0.001
PPO (W)	133 (40)	148 (46)	140 (43)	155 (47)	0.99	<0.001
HR at peak exercise	136 (22)	136 (26)	139 (16)	139 (21)	0.94	0.97
HR at 30% of PPO	94 (15)	93 (15)	95 (15)	91.2 (13)	0.30	0.92
HR at 70% of PPO	124 (16)	122 (23)	123 (17)	116.4 (16.4)	0.31	0.85
SBP at peak exercise	198 (27)	203 (27)	202 (27)	197 (31)	0.18	0.94
DBP at peak exercise	79 (10)	85 (11)	85 (8)	81 (13)	<0.001	0.61
SBP at 30% of PPO	144 (20)	145 (24)	149 (24)	142 (20)	0.26	0.41
DBP at 30% of PPO	72 (9)	74 (9)	74 (7)	74 (7)	0.51	0.55
SBP at 70% of PPO	181 (23)	183 (20)	179 (13)	178 (13)	0.71	0.89
DBP at 70% of PPO	80 (12)	79 (11)	76 (6)	76 (7)	0.84	0.91
LVEF (%)	57.6 (7.2)	59.3 (6.5)	58.2 (5.0)	62.4 (5.1)	0.20	<0.001
P_ET_CO_2_ at 30% of PPO (mmHg)	39.1 (3.1)	38.8 (3.7)	38.8 (3.3)	38.8 (3.4)	0.71	0.78
P_ET_CO_2_ at 70% of PPO (mmHg)	40.7 (4.6)	39.9 (3.3)	39.9 (4.1)	40.0 (4.1)	0.32	0.50
Medication						
Aspirin, *n* (%)	18 (100)	–	15 (88)	–	–	0.44
DAPT, *n* (%)	10 (56)	–	11 (65)	–	–	0.84
RAAS inhibitors, *n* (%)	9 (50)	–	11 (65)	–	–	0.59
Beta‐blockers, *n* (%)	11 (62)	–	12 (71)	–	–	0.81
CCB, *n* (%)	5 (28)	–	4 (24)	–	–	>0.99
Diuretics, *n* (%)	2 (11)	–	1 (6)	–	–	>0.99
Lipid lowering therapy, *n* (%)	18 (100)	–	17 (100)	–	–	0.87
Antidiabetic, *n* (%)	4 (28)	–	4 (24)	–	–	>0.99

Abbreviations: CCB, calcium channel blockers; DAPT, dual antiplatelet therapy; DBP, blood pressure diastolic; GDS, Geriatric Depression Scale; HR, heart rate; M, mean; MMSE, mini‐mental state evaluation; Peak V̇O_2_, peak oxygen consumption; PetCO_2_, partial pressure of end tidal CO_2_; PPO, peak power output; RAAS, renin–angiotensin–aldosterone system; SBP, blood pressure systolic; SD, standard deviation.

### 3.2 Cognitive performance during exercise

#### 3.2.1 Error rate

**FIGURE 1:**
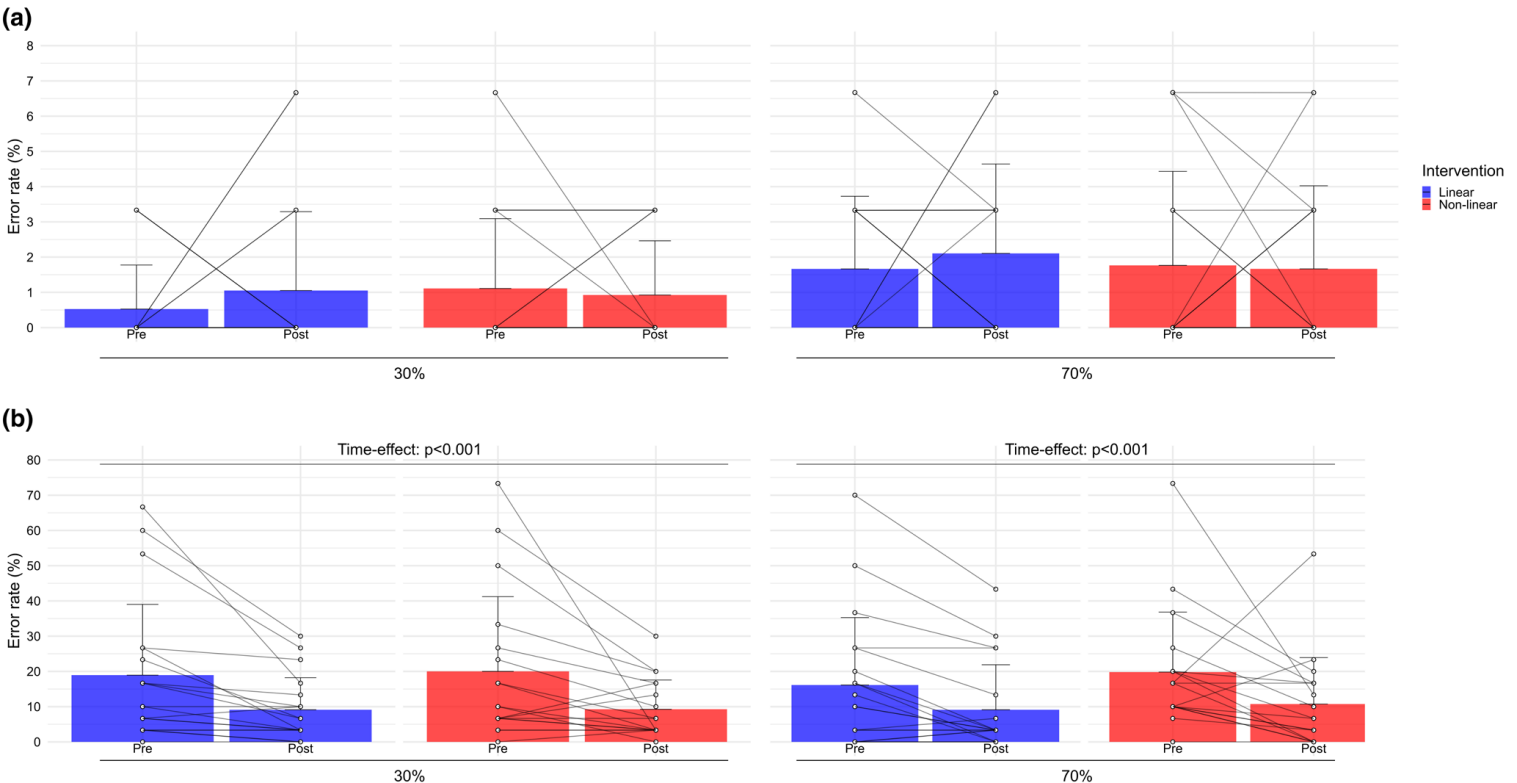
Means and standard deviations for Stroop task error rate pre and post LN and NLP interventions for 30% and 70% of PPO. (a) Error rate for naming condition. (b) Error rate for executive condition.

Figure 1 shows error rate as a function of time, intensities, groups, and Stroop task conditions. The two‐way ANOVA showed a main time‐effect: error rate was higher in pre training compared to post training for executive condition at 30% of PPO, *F*(1,33) = 14.83, *p* < 0.001, *η* ^2^ = 0.31, and at 70% of PPO, *F*(1,33) = 10.67, *p* < 0.001, *η* ^2^ = 0.24. There was no significant interaction‐effect for naming and executive conditions at 30% and 70% of PPO (all *p* > 0.05).

#### 3.2.2 Reaction time

**FIGURE 2:**
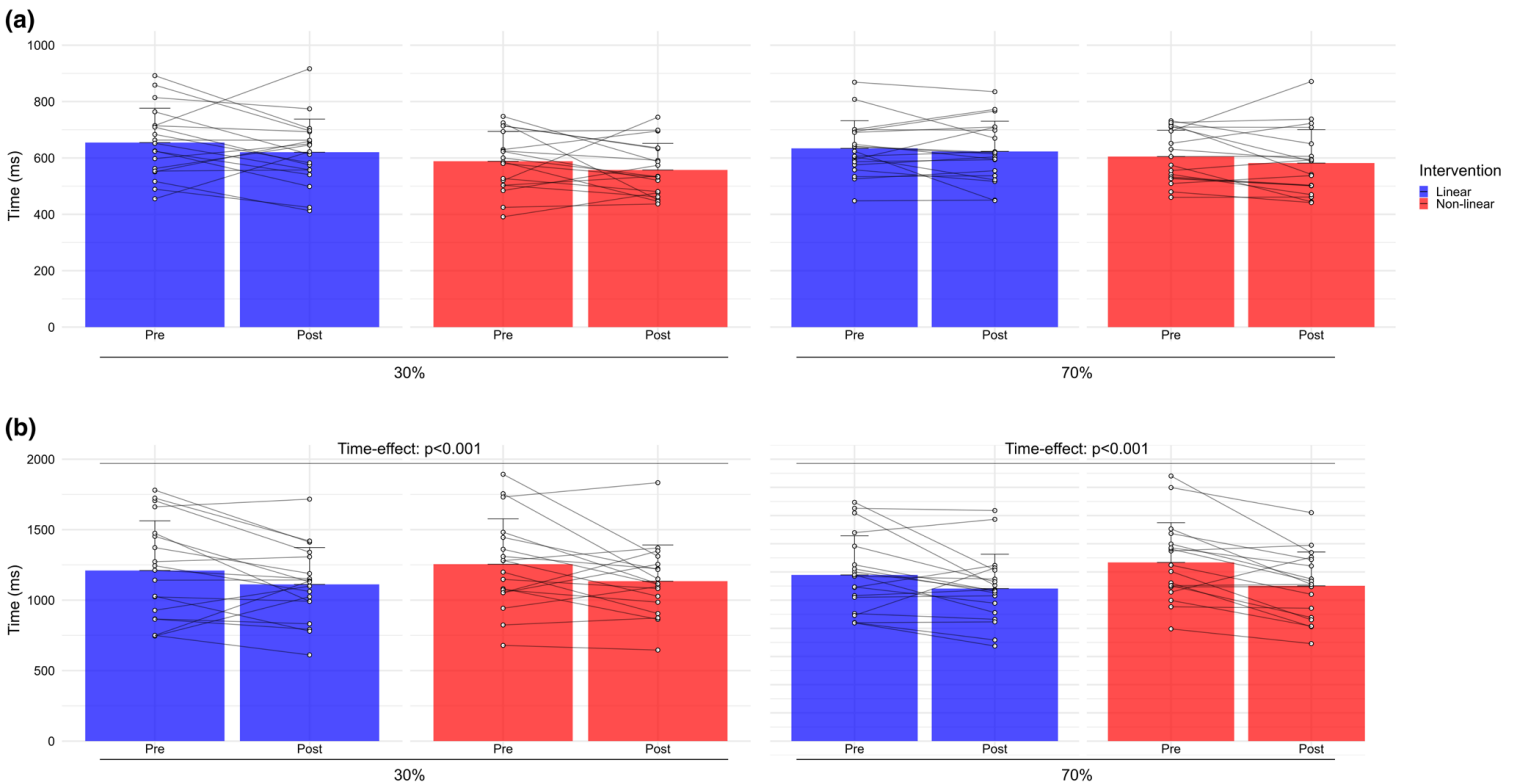
Means and standard deviations for Stroop task reaction time pre and post LN and NLP interventions for 30% and 70% of PPO. (a) Reaction time for naming condition. (b) Reaction time for executive condition.

Figure 2 shows reaction time as a function of time, intensities, and Stroop task conditions. We found a main time‐effect: reaction time was slower in pre training compared to post training for executive conditions at 30% of PPO, *F* (1, 33) = 7.22, *p* = 0.01, *η* ^2^ = 0.18, and at 70% of PPO, *F* (1, 33) = 14.69, *p* < 0.001, *η* ^2^ = 0.31. There was a main group‐effect for reaction time in the naming condition at 30% of PPO, *F* (1, 33) = 4.94, *p* = 0.03, *η* ^2^ = 0.13. Reaction time was slower in the LP compared to NLP. There was no significant interaction between time and groups for naming and executive conditions at 30% and 70% of PPO.

### 3.3 Cerebral oxygenation and perfusion

**FIGURE 3:**
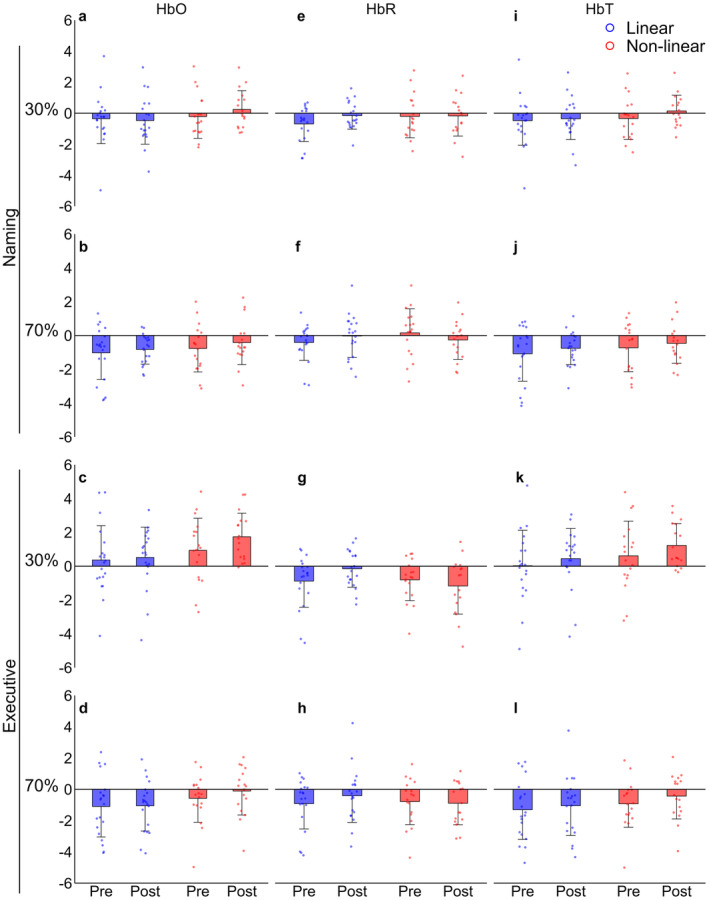
Cerebral oxygenation and perfusion (HbO: a, b, c, d; HbR: e, f, g, h; HbT: i, j, k, l) before and LP (○) and NLP (□) according 30% (a, e, i, c, g, k) and 70% (b, f, j, d, h, l) of PPO in naming (a, e, i, b, f, j) and executive (c, g, k, d, h, l) conditions in CHD patients. CHD, ccoronary heart disease; HbO, oxyhemoglobin; HbR, deoxyhemoglobin; HbT, total hemoglobin; LP, linear periodization; NLP, nonlinear periodization; PPO, peak power output.

There was no significant main effect of time or group for [HbO], [HbR], and [HbT] in all task conditions and intensities. There was no significant main effect of time or group for [HbO], [HbR], and [HbT] in all task conditions and intensities. There was no significant interaction between time and groups for naming and executive conditions at 30% and 70% of PPO (Figure 3).

## 4 DISCUSSION

To the best of our knowledge, this is the first study investigating the effects of two periodized training protocols on cognitive functions and cerebrovascular components during an acute bout of aerobic exercise at different intensities in CHD patients. First, we found that cognitive performances were improved after the 3‐month training intervention regardless of the type of training periodization and the exercise intensity. However, variations of cerebral hemodynamic response ([HbO], [HbR], and [HbT]) remained stable between the different types of training protocol, intensity, and time. This suggests that an increase in executive performance is not necessarily concomitant to an increase in cerebral perfusion after an exercise intervention.

### 4.1 Effects of exercise training on cognitive function: Effect of acute exercise

Both reaction time and error rate improved for the executive condition after training interventions, regardless of exercise intensity and training protocol. Improvement in cognitive performance is consistent with the literature in both chronic and acute exercise (Ando, 2016; Angevaren et al., 2008; Basso & Suzuki, 2017; Bherer et al., 2013; Labelle et al., 2013). Reaction time and executive performances are well known to improve after an exercise program that increase significantly cardiorespiratory fitness levels in older adults (Bherer et al., 2013; Dupuy et al., 2015). The relationship between the acute effects of exercise and cognitive performances may be explained by Dietrich's reticular‐activating hypofrontality (Dietrich, 2006; Dietrich & Sparling, 2004). This theory suggests that while exercising at high intensity, the limited brain resources, such as glucose and oxygen, are monopolized by the brain areas related to motor movement and coordination. Thus, these resources are switching from prefrontal areas in charge of high cognitive processing to primary motor cortex, cerebellum, etc., which are related to motor functions. This shifting of finite resources between brain areas provokes a deactivation and hypoperfusion in the prefrontal cortex. As a result of this hypoperfusion, oxygenated blood and cerebral activation enhance again in prefrontal cortex. From this perspective and since reaction time also include motor ability (Ando et al., 2009), this could explain why a faster reaction time was observed. This cognitive function is also known to improve with exercise, simultaneously with higher cardiorespiratory fitness (Desjardins‐Crépeau et al., 2014, 2016; Tam, 2013).

### 4.2 Cerebral oxygenation and cognitive function

Findings for cerebral oxygenation variations in the left prefrontal cortex were not in accordance with our hypothesis. Variations of [HbO], [HbR], and [HbT] remained unchanged after the exercise intervention, regardless of the cognitive task conditions, intensities, and training protocol. At low‐to‐moderate intensities (40%–60% of PPO), [HbO] tend to elevate especially while performing an executive task (Ando et al., 2009), while a decrease in [HbO] occurs generally at higher intensities (≥80% of PPO) in healthy population (Ando, 2016). However, a reduction of variations of cerebral oxygenation was not concomitant with impaired executive performances. This could be explained by the level of physical activity of our participants. Reduction in cerebrovascular reactivity, defined as change in cerebral blood flow induced by a stimulus, in the prefrontal cortex while performing high intensity exercise is more important in inactive populations (Rooks et al., 2010). In other words, cerebral oxygenation and executive performance of individuals with a higher level of fitness (Labelle et al., 2013) are less affected negatively by HI than those with a lower fitness level (Brisswalter et al., 2002). Cerebral oxygenation and perfusion are not the only hypothesis explaining cognitive improvement usually observed after an exercise program. In acute exercise, cerebral oxygenation is associated with cognitive performance (Ando, 2016; Mekari et al., 2015), but other components such as growth factor, neurogenesis, or lactate concentration are also associated with cognitive gain with chronic exercise, implying that cognitive benefits could be due to combined effects of overall mechanism (Norman et al., 2018; Quigley et al., 2020).

### 4.3 Fitness and cognitive function

At behavioral level, healthy individuals with higher peak V̇O_2_ had greater cognitive performance regardless of age in acute exercise session (Labelle et al., 2013). Since aerobic fitness is known to play a major role in cognition in the aging population (Bherer et al., 2013, 2021; Dupuy et al., 2015), the high fitness level of our CHD patients could partially explain why no differences between groups were observed for cognitive performance and for cerebral perfusion. In CHD individuals, higher baseline peak V̇O_2_ is an important predictor of nonresponse of peak V̇O_2_ improvement (De Schutter et al., 2018). Peak V̇O_2_ for CHD patients was considered higher than the predicted value for age‐matched healthy individuals meaning that cognitive performances and cerebral oxygenation could be influenced by fitness level in this clinical population (Colcombe et al., 2003; Gayda et al., 2017; Guazzi et al., 2016; Ruscheweyh et al., 2011). In another study, NLP training increased peak V̇O_2_ by 15.8% (de Macedo et al., 2012). In our study, there was a modest improvement of 6.6% for NLP and 7.5% for LP, which could explain why no changes in cerebral oxygenation and perfusion were found during the acute bout of exercise. Further details are available in Appendix S1.

### 4.4 Clinical relevance of an acute bout of exercise

Testing the effect of a chronic exercise program on cognition and cerebral hemodynamics during an acute bout of exercise at higher intensities (≥80% and over) would provide enlightenments on the relationship between fitness, cognition, cerebral oxygenation, and their relation with CBF in CHD population. Testing this protocol among CHD patients at higher risk of heart failure could also be relevant. Koike et al. (2008) used maximal exercise as a proxy to establish a clinical significance of cerebral variations in [HbO] to determine which CHD patients are at higher risk to have a second cardiac event. Lower [HbO] variations during submaximal exercise predicted future cardiac events among CHD patients and cerebral oxygenation was a stronger predictor than others cardiac indexes such as peak V̇O_2_ and left ventricular ejection fraction (LVEF). From this perspective, other studies could help clarify if an acute bout of exercise could potentially be used to diagnose CHD patients at higher risk of cognitive decline, which could not always be detected at rest using neuropsychological assessments (Brugniaux et al., 2014). This method could also help clarify if cerebrovascular markers are responsive to exercise and if they are related to cognitive performance in this population.

### 4.5 Limitations

Limitations of the study are details in Appendix S1. Despite the absence of conjunction between cerebral oxygenation and cognition, our results suggest that both exercise‐training programs were successful in improving cognition among CHD patients during acute exercise. In CHD individuals, more variations in the exercise intervention (NPL) were not necessary to show greater cognitive performances. In the context of cognition, the effects of an acute aerobic exercise could reflect critical situations of daily life such as housekeeping or walking (Ainsworth et al., 2000). These results suggest that chronic exercise, independently from its variations, could help to reduce the impact of CHD on daily functional status requiring cognition. Further studies are required in higher‐risk cardiac patients and low cardiorespiratory fitness to clarify which type of exercise training is the most efficient to prevent any CHD related cognitive consequences.

## AUTHOR CONTRIBUTIONS

B.B. drafted the manuscript. All authors contributed to the interpretation of results, revising the manuscript and approved the final version of the manuscript and agree to be accountable for all aspects of the work. M.B, L.B., M.G, J.T., A.N., and M.J. conceived and designed the study. M.G. and M.B completed data collection. The study was performed at the Cardiovascular Prevention and Rehabilitation Center (EPIC) of the Montreal Heart Institute. B.B., L.B., T.V., M.B., M.G., and L.B. analyzed the data. All interpreted the data and revised the manuscript. All designated as authors qualify for authorship, and all those who qualify for authorship are listed.

## FUNDING INFORMATION

No funding information provided.

## CONFLICT OF INTEREST STATEMENT

Authors declare no conflict of interest to declare.

## ETHICS STATEMENT

We confirm that we have read the Journal's position on issues involved in ethical publication and affirm that this report is consistent with those guidelines.

## Supporting information


Appendix S1.

## Data Availability

Data requests may be sent to Dr. Maxime Boidin at the Institute of Sport of Manchester Metropolitan University (m.boidin@mmu.ac.uk).
