# Supplementary material for: Impact of two different periodized aerobic training on acute cerebrovascular response and cognitive performance in coronary heart disease patients
Source: Physiol Rep. 2025 Feb 4;13(3):e70211. doi: 10.14814/phy2.70211 (PMC11792991; doi:10.14814/phy2.70211)
Supplement: Supplementary file 1 — Appendix S1. [file PHY2-13-e70211-s001.docx]

Previous studies showed a reduced [HbO] in cardiovascular disease population during exercise compared to healthy controls and a positively association between [HbO], LVEF and $\dot{V}$O_2_peak (Gayda et al., 2017, Koike et al., 2004, Koike et al., 2006). That is, it has been demonstrated that cardiac patients with lower LVEF and $\dot{V}$O_2_peak had lower [HbO] during acute exercise (Koike et al., 2006). Cardiovascular disease participants who had a lower $\dot{V}$O_2_peak and lower LVEF also had a reduced cardiac output, which could lead to lower blood volume, hypoperfusion and consequently, reduced variations of cerebral [HbO] (Nagayama et al., 2007, Rooks et al., 2010). In our study, LVEF also slightly improved regardless of exercise group after the intervention program but remained in the normal range values. The modest improvement of $\dot{V}$O_2_peak and LVEF could explain why better executive function during an acute bout of exercise was not concomitant with an increase of cerebral perfusion. During an acute session of exercise at 70% of PPO, CHD individuals had larger negative variations of [HbT] in left prefrontal cortex compared to healthy match subjects (Bérubé et al., 2021) even if CHD patients had preserved LVEF (≥ 40 %). However, this result was not concomitant with an impaired executive performance compared to control group. In this study, a better executive performance was observed without any change of cerebral perfusion and oxygenation after the intervention. Taking together, these results suggest that the intensity used in both studies could have been too low to detect any changes in dependant variables of interests and the effects of exercise on cognition target different pathways for chronic and acute exercise.

This study was the second to measure cognitive performances during an acute bout of exercise in CHD patients (Bérubé et al., 2021) but the first using two periodized training protocols to measure cognitive performances during an acute bout of exercise in this cardiac population. Participants were selected based on strict criteria including a minimal risk to engage in an exercise session and stable CHD. The results should be considered within its limitations despite those several strengths and novel insights. We carefully chose 70 % of PPO to ensure their capacity to perform the complete duration of the acute bout of exercise. This intensity level may be too low to detect any changes in cerebral oxygenation and higher intensities could have been used considering their higher fitness level (Ando, 2016, Bhambhani et al., 2007, Labelle et al., 2013, Mekari et al., 2015). Further studies should consider higher intensity than 70 % of PPO during acute bout of exercise because it could provide better insight on cerebral oxygenation and on the measurements of optimal types of training. Moreover, chronic health condition of this sample could partly explain why no improvements were observed on cerebral oxygenation (Fu et al., 2013, Moriarty et al., 2020). First, the understanding of the relationship between cerebrovascular and cognitive components is limited to prefrontal region only. Adding more cerebral areas could provide insights on non-executive components results and on how cerebral oxygenation in other areas reacts to acute exercise. Although we applied a filter to attenuate the risk of skin blood volume contamination, a better control for skin blood volume on forehead could have been performed to reduce interference with cortical blood volume measurements. Finally, our CHD patients were relatively fit before they started the chronic exercise program (Guazzi et al., 2016).
